# Ecological effects of stress drive bacterial evolvability under sub-inhibitory antibiotic treatments

**DOI:** 10.1038/s43705-022-00157-w

**Published:** 2022-09-02

**Authors:** Marie Vasse, Sebastian Bonhoeffer, Antoine Frenoy

**Affiliations:** 1 Institute for Integrative Biology, ETH Zürich, Zurich, Switzerland; 2 Université Grenoble Alpes, CNRS UMR 5525 Grenoble, France

**Keywords:** Evolution, Antibiotics

## Abstract

Stress is thought to increase mutation rate and thus to accelerate evolution. In the context of antibiotic resistance, sub-inhibitory treatments could then lead to enhanced evolvability, thereby fuelling the adaptation of pathogens. Combining wet-lab experiments, stochastic simulations and a meta-analysis of the literature, we found that the increase in mutation rates triggered by antibiotic treatments is often cancelled out by reduced population size, resulting in no overall increase in genetic diversity. A careful analysis of the effect of ecological factors on genetic diversity showed that the potential for regrowth during recovery phase after treatment plays a crucial role in evolvability, being the main factor associated with increased genetic diversity in experimental data.

## Introduction

A key aspect of the fight against antibiotic resistance concerns the origin of the genetic innovations generating resistance phenotypes. The generation of genetic diversity on which natural selection can act being at the heart of evolutionary biology, the genetic and environmental factors influencing mutagenesis have been under close scrutiny. Beyond the well-known role of mutator alleles which constitutively increase the mutation rate of a genome [1], it has been suggested that many abiotic stresses, including sub-inhibitory antibiotic treatments, can phenotypically and transiently increase mutation rate [2–6]. This implies that low doses of antibiotics would not only select for pre-existing resistance alleles [7], but could also accelerate the generation of random genetic diversity, including rare antibiotic resistance alleles [8].

The actual –realized– genetic diversity, however, is not only determined by mutation rate. Ecological factors, and population size in particular, further plays a crucial role [9, 10]: they impact the total absolute number of mutant genotypes accessible to natural selection, which determines the adaptive potential of a population. In the context of antibiotic resistance, this is for example reflected by the evolution of antibiotic tolerance often preceding resistance [11]. Therefore, understanding the role of antibiotics on the ability of a population to generate genetic diversity, and thus on evolvability, critically relies on combining the study of the physiological effects of antibiotics at the molecular level (e.g. mutagenesis) with the study of their ecological impacts. The need for such integration of mutagenesis with ecological factors is well exemplified by our previous work [12], in which we exhibited the scenario of a treatment increasing mutation rate and decreasing genetic diversity. Here, beyond this previous anecdotal finding concerning one particular antibiotic at a specific concentration, we aim at systematically exploring the effect of antibiotic stress on the generation of genetic diversity in bacteria.

Properly isolating and quantifying mutation rates, doubling times and death rates is not always possible. The estimation of mutation rate in particular brings intrinsic difficulties, because it relies on sophisticated mathematical models making strong biological assumptions (including the absence of cell death) that are not always pertinent, especially under stress [12]. Instead, we directly address the question of the effect of stress on the generation of genetic diversity, and thus on evolvability, which is defined as the capacity of a population to generate adaptive genetic diversity, and therefore to evolve by natural selection. More specifically, while the concept of evolvability encompasses both the quantitative genetic diversity generated by a population and the fraction of such diversity which is adaptive [13], we here focus on the former. To quantify the effect of stress on genetic diversity in fluctuation test-like experimental setups, we suggest a simple and easy-to-collect metric which does not rely on mutation rate computation. This metric directly relies on the observed number of mutants towards a neutral arbitrary phenotype. It is calculated as the ratio of the observed number of mutants in treated populations to the observed number of mutants in untreated controls (see *material and methods* section 5.3 for details). While the observed number of mutants is not exactly the mutation supply, because the same mutation event can give several clones in the final population, both variables are closely related and the former can be directly measured in practice (see section 5.4 for further discussion on potential limitations of this metric).

To assess the role of antibiotic treatments on the generation of genetic diversity in bacteria, we collected raw data from primary literature on the effect of antibiotic treatments on mutation rates using fluctuation test and its variants, and conducted a meta-analysis with emphasis on the effect of treatments on both mutation rate and population size. Additionally, we performed our own set of experiments for the most commonly studied antibiotics, to mitigate the difficulty of comparing data from different labs in meta-analyses. We show that the decrease in population size due to the treatments often cancels the potential increase in mutation rate, resulting in no overall increase of genetic diversity. We further combine the experimental data with simulations to explore the ecological conditions in which antibiotic treatments may still foster the generation of genetic diversity.

## Results

**Fig. 1:**
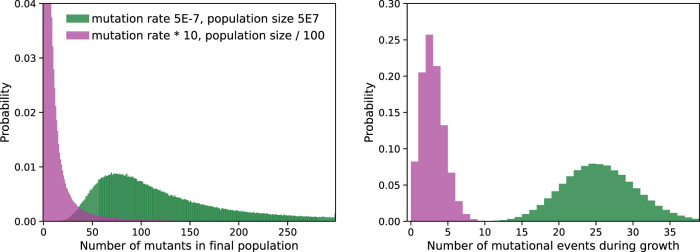
Change in genetic diversity due to a hypothetical treatment that increases mutation rate (x10) but decreases population size (/100). The left panel shows the distribution of the number of mutants in the final population for 1000 simulations. The right panel shows the distribution of the number of mutational events in the same simulations. The number of mutational events gives a more precise estimation of genetic diversity, because a single mutational event can give several times the same mutant in the final population. The number of (non-unique) mutants, however, can be directly measured experimentally.

Our work stems from the simple intuition that an antibiotic treatment which increases mutation rate by tenfold but decreases population size by 100-fold is not likely to increase genetic diversity (as for example noted by [14], and as suggested by our previous *n* = 1 observation [12]). This is consistent with the broad definition of mutation supply as the overall product of mutation rate and population size (e.g. [15, 16]), and with the more specific analysis of the factors determining the rate of evolution in a simple population genetics model by [17]. We confirmed this intuition with stochastic simulations of the arisal of neutral mutations in a population subject to simple ecological forces (neutral birth and death) with a constant mutation rate. These simulations mimic the experimental conditions of the Luria and Delbrück fluctuation test (see material and methods section 5.5). We found that, in this simple scenario, the treatment *decreases* the generated genetic diversity (i.e. the total number of mutants and of unique mutational events, *p* < 10^−9^), Fig. 1 (bacteriostatic treatment) and Fig. S3 (bactericidal treatment).

This observation questions whether the antibiotic treatments reported in the literature to increase mutation rate also have an effect on population size, and whether this effect may outbalance the reported increase in mutation rate, resulting in unchanged or even decreased genetic diversity. To address this question, we re-analyzed the raw data from ten published studies on the effect of sub-inhibitory antibiotic treatments on mutation rate (Table 1, [12, 18–26]), that we complemented with our own experimental data. These studies constitute all the recent (posterior to 2008) literature for which we could obtain raw data that compute bacterial mutation rate under antibiotic stress using a protocol based on the historical fluctuation test [27]. The standard modern use of fluctuation test is the quantification of mutation rate using the observed number of mutants towards a neutral phenotype during a single overnight growth of initially isogenic bacterial populations, which allows to uncouple the effects of mutagenesis from the effects of selection. Here, we gathered and produced data from fluctuation tests performed in different environments, allowing to infer the effect of the environment – and more specifically of antibiotic stress – on mutation rate. Although it may not permit to study the effects of stress on mutation spectrum (see *material and methods* section 5.3), this test is the *de facto* standard for mutation rate estimation. It is very closely related to Ames test, frequently used to assess mutagenecity of chemicals in a more medical context [28].

**Table 1:** Experimental data analyzed in this work.

Article	Protocol	Variable	Antibiotic (µg/mL)	Bacteria	Marker
Baharoglu et al. [18]	FT	frequency	Ampicillin (0.05), Ciprofloxacin (0.05), Chloramphenicol (0.15), Gentamicin (0.1), Kanamycin (0.2), Mitomycin C, Neomycin (0.1), Rifampin (0.05), Spectinomycin (0.2), Tetracycline (0.15), Tobramycin (0.1), Trimethoprim (0.05)	*E. coli, V. cholerae*	rifR
Cortes et al. [19]	FT	rate	Chloramphenicol (3.0), Erythromycin, (0.09), Penicillin (0.024)	*S. pneumoniae*	rifR, optoR
Dapa et al. [20]	FT	frequency	Mitomycin C (1.0)	*E. coli*	rifR
Frenoy et al. [12]	FT	rate	Kanamycin (3.0),Norfloxacin (0.05)	*E. coli*	rifR
Giroux et al. [21]	FT	frequency	Trimethoprim (0.04)	*E. coli*	tetR
Hocquet et al. [22]	FT	frequency	Metronidazole (50.0)	*P. aeruginosa*	cipR, amkR
Jara et al. [23]	Regrowth	frequency	Ciprofloxacin (0.0625), Colistin (2.5), Meropenem (0.125), Tetracycline (1.5)	*A. baumannii*	rifR
Mo et al. [24]	FT	rate	Ampicillin (2.0), Ciprofloxacin (0.01), Mitomycin C (1.0), Nitrofurantoin (2 − 4), Novobiocin (16.0), Streptomycin (2.0), Trimethoprim (0.032)	*E. coli*	rifR
Rodríguez-Rojas et al. [25]	Regrowth	rate	Ampicillin (3.2), Ciprofloxacin (0.05), Kanamycin (1.6)	*E. coli*	rifR
Torres Barceló et al. [26]	FT	rate	Ciprofloxacin (0.048)	*P. aeruginosa*	rifR
This study	FT	rate	Ampicillin (1.0, 3.2), Ciprofloxacin (0.005), Chloramphenicol (0.15, 1.5), Kanamycin (1.6), Mitomycin C (1.0), Nalidixic acid (1.0), Norfloxacin (0.005, 0.05), Streptomycin (5.0), Tetracycline (0.15), Trimethroprim (0.005, 0.05)	*E. coli*	rifR

Origin, protocol, reported variable, antibiotic and its concentration, bacterial species, and marker used to score mutants. The protocol can be “FT”, indicating a standard fluctuation test, or “Regrowth”, indicating a modified fluctuation test in which cultures recover in fresh antibiotic-free medium following antibiotic exposure. The reported variable indicates whether the original study reported mutation rates or mutation frequencies. In both cases, we use the raw data for mutant counts, population size, and plating fraction to analyze all datasets with the same methods.

**Fig. 2:**
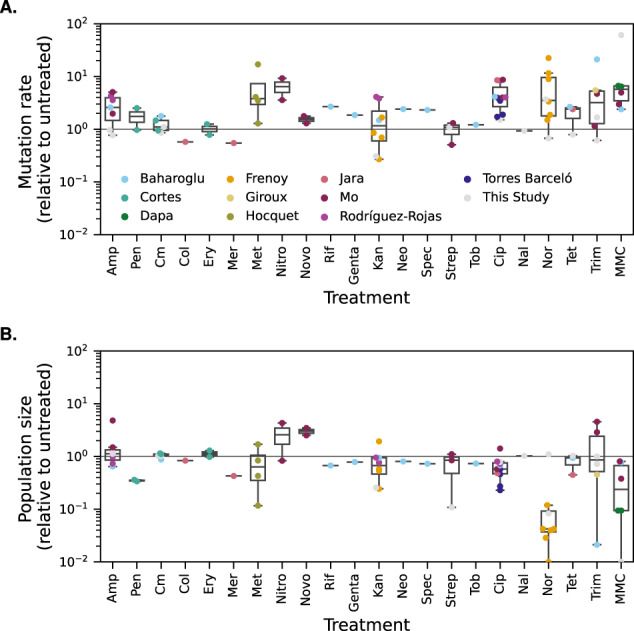
Effect of antibiotic treatments on estimated mutation rate and population size. Different antibiotics are shown in different columns on the x axis, ordered by class. Colours indicate different studies (ten datasets from the literature plus our data). A single study may comprise several data points associated with the same antibiotic because several doses were tested or because several biological replicates (each of them comprising several parallel replicate populations) were performed. **A** Effect of antibiotic treatments on estimated mutation rate, under the assumption that there is no cell death. All mutation rates are relative to the untreated control (rel. NT, horizontal line y = 1). The mutation rates were estimated using rSalvador [29]. **B** Effect of antibiotic treatments on population size. All population sizes are relative to the untreated control (horizontal line y = 1). Each data point represents the average final population size in several parallel replicate populations treated with a specific dose of a given antibiotic.

We first evaluated the effect of the antibiotic treatments on both computed mutation rates and population sizes. Estimating mutation rates with a widely used modern method (rSalvador [29], ignoring cell death), we confirmed a systematic increase in computed mutation rates in the presence of sub-inhibitory doses of antibiotics in both data from the literature and our own data (geometric average of the relative increase over all data points: 2.33 fold, significantly higher than 1 (Wilcoxon signed-rank test *p* = 2.32 × 10^−9^), Fig. 2A). However, at these concentrations reported to increase mutation rate, antibiotic treatments overall concomitantly decreased population size (geometric average fold change 0.51, *p* = 1.34 × 10^−5^, Fig. 2B).

**Fig. 3:**
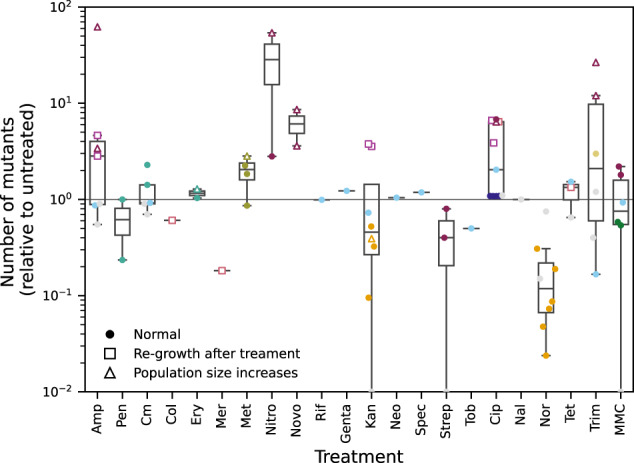
Effect of antibiotic treatments on genetic diversity. The median of the raw number of mutants detected on selective medium among several replicate populations is used as an approximation of genetic diversity. This quantity is relative to the untreated control (horizontal line y = 1). The colours correspond to different studies and are the same than on Fig. 2. Open squares indicate the use of a modified fluctuation assay protocol where treated cells are regrown in fresh antibiotic-free medium before being plated. Open triangles indicate the particular situation where antibiotic treatment increases population size. All other data points (standard fluctuation assay, no increase in population size) are shown as filled circles.

We then addressed the central question of the overall outcome of decreased population size and increased mutation rate on genetic diversity. Using our proposed metric of generation of genetic diversity in a bacterial population, we found that the effect of antibiotic treatments on the number of mutants for the neutral phenotype of interest is more equivocal, with great variation both between and among antibiotics (Fig. 3). Interestingly, the data points indicating higher genetic diversity, i.e. more mutants compared to the untreated baseline, mostly belonged to a small subset of studies. We therefore wondered whether systematic differences between the experimental conditions could account for this finding. We found that the data points for which genetic diversity increased were (1) those originating from studies using a variant of the fluctuation test protocol in which populations recover in fresh antibiotic-free medium after antibiotic exposure, resulting in regrowth after treatment, and (2) those in which population size was found to increase in the treated populations compared to the untreated.

Integrating these factors into our analysis of the effect of antibiotic treatment on genetic variability, we found that data points associated with regrowth after treatment or with increased population size have a significantly increased genetic diversity relative to untreated (geometric average fold change in genetic diversity 4.00, significantly higher than 1, *p* = 9.02 × 10^−4^ in Wilcoxon signed-rank test). Conversely, data points from standard fluctuation assay protocol show a moderate decrease in genetic diversity (geometric average fold change in genetic diversity 0.68, low evidence that lower than 1, *p* = 0.042 in Wilcoxon signed-rank test). The difference between the genetic diversity for the data points from the two categories is highly significant (Wilcoxon signed-rank test, *p* = 1.77 × 10^−6^).

The effect of regrowth after exposure to antibiotic stress can be understood in the light of the idea that genetic diversity depends on both the number of genome replications and on mutation rate. The additional divisions permitted by regrowth can then compensate for the initially reduced population size due to treatment. We confirmed this effect detected in the meta-analysis using simulations of treatments with and without regrowth (Fig. S4, S5): genetic diversity is increased (both compared to untreated controls and to treatment without regrowth) for a treatment followed by a regrowth phase.

## Discussion and conclusion

Sub-inhibitory stresses are pervasive in nature, and non-lethal concentrations of antibiotics in particular are commonly found in soils, rivers, and drinking water, in addition to treated patients [30]. As a consequence, bacteria are frequently exposed to low amount of antibiotics, and these stressed populations are often thought to drive the evolution of stress-resistance phenotypes [31]. A quantitative understanding of the generation of genetic diversity under stress is thus pivotal to the study of adaptation in microbes.

In this work, we show that the ecological effects of stress change the relationship between mutation rate and genetic diversity, making mutation rate a poor predictor of the generation of genetic diversity and thus of evolvability. Our reanalysis of the datasets obtained from ten published works shows that sub-inhibitory antibiotic stress generally decreases rather than increases genetic diversity. A notable exception to this result comes from protocols designed to let cells regrow after exposure to antibiotic stress, to allow for cell recovery from antibiotic-triggered filamentation before assessing their phenotype [32]. Regrowth should however not be merely regarded as a peculiarity of this protocol. Indeed, in natural settings, antibiotic concentrations are frequently heterogeneous in time and space, and pathogens as well as commensals in a complex biotic environment such as the gut or soil microbiomes may be subject to cycles of stress treatment and regrowth. In such cases, we expect that the potential for regrowth, and thus the genetic diversity, will depend on the presence of and relationship to antagonistic organisms in the same ecological niche and on the antibiotic spectrum.

The measure of the generation of genetic diversity we propose here is particularly suitable to analyse experiments with regrowth, as regrowth invalidates the mathematical model behind mutation rate computations from a fluctuation test and changes the relationship between mutation rate and genetic diversity. As our measure encompasses both mutational and ecological forces, it remains valid in presence of cell death which biases the estimations of mutation rates with standard tools [12]. Our work shows that even if it would be possible to accurately compute mutation rate in populations treated with antibiotics or other stressors, this rate would not be the relevant measure to evaluate the generation of genetic diversity.

Studies of microbial mutation rates demonstrated that both genetic and environmental factors can affect mutation rate [1, 33, 34]. Our observation that an increase in mutation rates is often compensated by a decrease in population size echoes this literature in several ways. Manipulating population size by varying the concentration of glucose in fluctuation tests, Krašovec, Knight and collaborators found that mutation rate is lower when population size is higher [35], suggesting that population density exerts an environmental effect on phenotypic (transient) mutation rate. Interestingly, Sung, Lynch and collaborators made parallel observations at a different scale: studying the link between the constitutive mutation rate of a species and its typical effective population size, they found that those are negatively correlated. This led them to formulate the drift-barrier hypothesis for the evolution of mutation rate [36], which suggests that DNA replication is always selected for highest accuracy, but that effective population size is a barrier to the efficiency of such selection. Said otherwise, this hypothesis implies that the mutation rate of a microbial species should not be considered as fine-tuned by evolution to ensure the best trade-off between maintaining genome integrity and generating genetic diversity. Instead, it proposes that natural selection overall selects for mutation rate as low as possible (consistently with the reduction principle [37, 38]), and that such limitation (*a*s low as possible) is a consequence of effective population size: natural selection can only act on fitness differences of the order of 1/*N*_*e*_, below which drift is dominant. As a consequence, mutation rate is predicted to be inversely correlated with population size among microbial species. Similarly, our observation could be thought in the framework of the drift-barrier hypothesis. While error-prone polymerases [39, 40] are often believed to be selected to generate more diversity under stress [41, 42], in the context of second-order selection for evolvability [43–45], an alternative reasoning could also hold here. Because these polymerases repair DNA damaged by various exogenous stresses, they could systematically act under conditions of reduced effective population size and higher genetic drift (random death), and thus not be as efficiently selected for accuracy. In other words, one could speculate that instead of being fine-tuned by evolution for increasing genetic diversity when needed, error-prone polymerases are selected for maximal fidelity, but that the efficiency of this selection is limited by the ecological conditions under which these polymerases typically act.

With mutation rate computation posing many theoretical challenges, we here proposed to estimate the generation of genetic diversity using a simpler and directly measured variable, *i.e*. the raw number of observed mutants for an arbitrary selectable phenotype in the treated population relative to the untreated controls. We conclude that, at the chosen concentrations, antibiotic treatments do not increase genetic diversity when the bacteria are not regrown after treatments. While mutation rate is a crucial variable for the understanding of the molecular effects of various stresses, we show here that it is a poor predictor of genetic diversity and thus of evolvability when stress also exerts ecological effects.

## Material and methods

### Meta-analysis of previous studies

We focused on studies posterior to 2008 (no more than ten years old at the beginning of this project) which quantified the effect of sub-inhibitory antibiotic treatments on bacterial mutagenesis. We were able to gather raw data for ten studies (see Table 1). For each study, we extracted the raw data of the number of mutants (toward a chosen neutral phenotype) and total bacterial densities both after antibiotic exposure and in untreated controls, and the fraction of population plated on selective medium to score mutants. This list of variables reflects important features of mutation rate estimation using the standard fluctuation test devised by S. E. Luria and M. Delbrück: the initial bacterial density of the founder population does not matter as long as it is small, and partial plating needs to be mathematically accounted for [46].

The following studies contain relevant data, but were excluded from the analysis despite matching our *a priori* inclusion criteria: [6, 47] (data available, but in a different representation or in a summary form); [5, 48–55] (raw data unavailable or no answer from the author who kept the raw data).

Summary statistics (fold change in mutation rate, population size and number of mutants relative to untreated control) for the analyzed dataset are available in supplementary materials (Fig. S6).

### Experimental detection of de novo mutations under sub-inhibitory antibiotic treatments

We performed a protocol closely inspired by the historical fluctuation test [27]. The aim was not only to add new data for the most studied antibiotics, but also to generate data for different antibiotics under strictly comparable conditions.

#### Culturing conditions

Using an overnight culture of *Escherichia coli* MG1655, we applied a strong bottleneck to initiate 12 parallel replicate populations. Specifically, we diluted the culture 10^5^ times and inoculated 10 *μ*L into 1 mL of fresh LB (lysogeny broth, Miller formulation) supplemented or not with antibiotics, in 13 mL tubes (Sarstedt, Germany). We used 20 antibiotic conditions with 12 replicates each for a total of 240 cultures (9 different antibiotics with several doses and untreated control, see Table 1). We incubated the cultures at 37 °C shaken at 300 rpm for 24 h.

#### Estimation of mutant frequencies

After 24 h of growth in the presence of the different antibiotic treatments, we estimated the number of de novo mutants by plating 200 μL of each culture onto LB agar supplemented with 100 μg/mL rifampicin (i.e. rifampicin resistance is the neutral phenotype chosen to score *de novo* mutations). We further plated serial dilutions of six randomly chosen populations for each treatment onto LB agar to estimate total bacterial densities.

#### Mutation rate

We estimated mutation rate in the datasets from the literature and in our own experiments using rSalvador v1.7 [29], with a correction for partial plating. This estimation does not account for potential bacterial death during the experiment.

### Proposed alternative metric of evolvability from these experimental data

In our attempt to integrate both mutational forces and ecological factors in the quantification of genetic diversity generated under stress, we propose to use the raw number of mutants towards the neutral phenotype of interest in treated populations, compared to the raw number of mutants in untreated controls.

This metric allows to directly measure resulting genetic diversity instead of aiming at isolating and independently quantifying these factors using a complex model. It has the additional advantage to be easy to collect in the lab.

Because the raw number of mutants is highly sensitive to jackpots, the arithmetic mean is not the most appropriate statistic to summarize this quantity over several replicates. Instead we use the median, which is not as sensitive to these extreme values. The measure we use and display on Fig. 3 is thus the ratio of the median (among the parallel replicate populations) of the number of mutants observed in treated conditions to the median of the number of mutants observed in non-treated conditions. A ratio higher than one indicates increased genetic diversity.

Each data point on Fig. 3 corresponds to a biological replicate of mutation rate estimation comprising several parallel replicate populations. Since the quantity computed and reported for each data point is a ratio (fold change compared to untreated) as explained above, we use geometric mean when combining all data points to compute the overall effects of antibiotic treatment.

### Assumptions and limitations of our proposed metric

#### Locus-specific and genome-wide diversity; mutation spectrum

As always in fluctuation test, global quantification of the generation of genetic diversity relies on extrapolation from a specific class of mutation on a specific locus conferring a selectable phenotype (such as the traditionally used rifampicin resistance conferred by mutations in rpoB). The underlying assumption for this generalisation is not that mutation rate is uniform over the genome, but rather that mutation rate is increased (by genetic or environmental factors) in a similar fashion at all positions on the genome.

It is however known that mutation spectrum may be affected by stress [34], and thus that different classes of mutations (eg indels vs substitutions or transitions vs transversions) may be differently affected by stress. Changes in genetic diversity as well as in mutation rate might thus not be adequately summarized by a single quantity.

Depending on the chosen marker, this protocol may therefore not allow to study the effect of stress on the mutation spectrum.

#### Intrinsic variability of the number of observed mutants

Due to the large effect of the timing of appearance of mutations on the final number of mutants in the fluctuation test, this method requires a relatively high level of replication (compared to traditional binomial or poissonian dilution-sampling-plating assays). This is the case for all methods derived from the fluctuation test.

#### Fitness effect of the mutation

To estimate mutation rate from fluctuation tests, the phenotype used to score mutants should be neutral. We used rifampicin resistance (conferred by substitutions in rpoB), which is frequently used in the literature. However, some rifampicin resistance mutations have been shown to confer a fitness defect in *E. coli* [56], potentially leading to biases in the estimate of mutation rate. Such biases make it hard to compare mutation rates reported from different investigators using from different makers. Here we systematically compare mutation rate or genetic diversity in a treated population with an untreated control using the same marker and the same protocol. This relative measure is more robust –although not necessarily immune– to biases induced by departure from neutrality.

#### Link between number of mutants and number of mutational events

The reproduction of mutants that emerged early in the culture implies that each observed mutant in the final population of a fluctuation test does not necessarily correspond to a unique mutational event and thus to a unique genotype. However, as we verified from simulations shown on Fig. 1, the number of mutational events is closely related to the number of observed mutants, and antibiotic treatment is unlikely to systematically bias the relationship between both variables.

#### Link between number of mutational events and number of unique genotypes

As explained two paragraphs above, the inability to consider a varying mutational spectrum is a general caveat of the fluctuation test, which scores specific mutations in a specific locus. But this may be even more important in the specific context of stress-induced mutagenesis, where the rate of different types of mutations may be differently altered by the antibiotic treatment.

In particular, the SOS-induced polIV has been reported to leave a very specific and restricted mutation spectrum, different from the one observed in normal, stress-free growth conditions [57]. Consequently, the same mutations may be sampled more frequently under stressed conditions, and thus the number of mutational events may not accurately reflect the number of unique alleles.

We should note that this effect would actually amplify the main effect we report in this work: not only we showed that sub-inhibitory antibiotic treatments overall decrease the number of mutational events, but additionally these events would be less likely to correspond to unique alleles.

#### Link between genetic diversity and evolvability

Evolvability is defined as the capacity of a population to generate adaptive genetic diversity, and therefore to evolve by natural selection. This can be decomposed in two parts: the quantity of genetic diversity generated, and the fraction of this diversity which is adaptive. This work only concerns the first part, and for example does not consider questions such as the fitness landscape under antibiotic stress.

### Stochastic simulations

Stochastic simulations are useful to give empirical support to seemingly intuitive arguments (such as a treatment which increases mutation rate by tenfolds and decreases population size by 100-folds ought to decrease genetic diversity), but also to properly formalize such arguments. They further permit to explore more complex scenarios for which intuition shows its limitations, such as the effect of death and the potential difference between bacteriostatic and bactericidal treatments.

Our simulations feature two populations: a wild-type and a mutant. Individuals grow, die, and mutate, at given rates. They are initialised with a small number of wild-type bacteria, and continue until total population size reaches a predefined threshold (carrying capacity). The number of mutants is recorded at the end of the simulation. This mimics the experimental conditions of the fluctuation test.

Our simulation method relies on Gillespie algorithm [58], and thus features discrete populations. This is necessary, as continuous deterministic simulations based on ODE would lead to biologically unrealistic situations such as the existence of 0.0001 mutants after a very short growth time.

The simulation scheme is represented graphically in supplementary materials (Fig. S2).

The source code of our simulation program is included in the data deposit. More details on the parameters are given in supplementary materials (Text S1).

## Supplementary information

Supplementary material

## Data Availability

All raw data, simulation programs, data analysis and visualisation programs and processed data are published under a Creative Commons licence, permitting a full reconstruction of the figures and reproduction of the statistical analyses. This dataset is publicly available on Zenodo: 10.5281/zenodo.4265430.
