# Supplementary material for: Ecological effects of stress drive bacterial evolvability under sub-inhibitory antibiotic treatments
Source: ISME Commun. 2022 Sep 2;2:80. doi: 10.1038/s43705-022-00157-w (PMC9723650; doi:10.1038/s43705-022-00157-w)
Supplement: Supplementary file 1 — Supplementary material [file 43705_2022_157_MOESM1_ESM.pdf]

Supplementary material for *Ecological effects of stress drive bacterial evolvability under sub-inhibitory antibiotic treatments*

Marie Vasse, Sebastian Bonhoeffer, Antoine Frenoy

Text S1: Simulated effects of treatments on genetic diversity

Simulation data were obtained using stochastic simulations of accumulation of a neutral mutant allele in a population subject to known ecological forces (neutral growth and death with maximal carrying capacity) and mutational forces (mutation rate per individual per division toward a neutral mutant genotype), mimicking the fluctuation test used to produce the experimental data. The precise model listing the implemented stochastic processes as well as a qualitative illustration are presented on Fig. S2.

Simulation data presented on Fig. 1 (main text), S3, S4, S5 were obtained with the following parameter values: we simulate populations inoculated with 100 bacteria (which limits the risk of stochastic extinction) which grow to a final population size of 5E7 bacteria in absence of antibiotic treatment (carrying capacity). Mutation rate for the trait of interest is 5E-7 per genome per division in absence of treatment.

For the simulations presented in Fig. 1 (main text), final population size is reduced to 5E5 and death rate is null, mimicking a purely bacteriostatic action of the antibiotic. Fig. S3 presents the results of similar simulations, but with an antibiotic that also has a bactericidal action with a death rate 0.5 relative to birth rate.

The effect of regrowth following treatment is represented on Fig. S4 and S5: after the treatment phase during which mutation rate is increased (x10) but final population size is lowered (/ 100) compared to untreated, a recovery phase is simulated in which population size reaches the same final value as in untreated conditions, with the same mutation rate value.

For each condition, 1000 replicate stochastic simulations were performed.

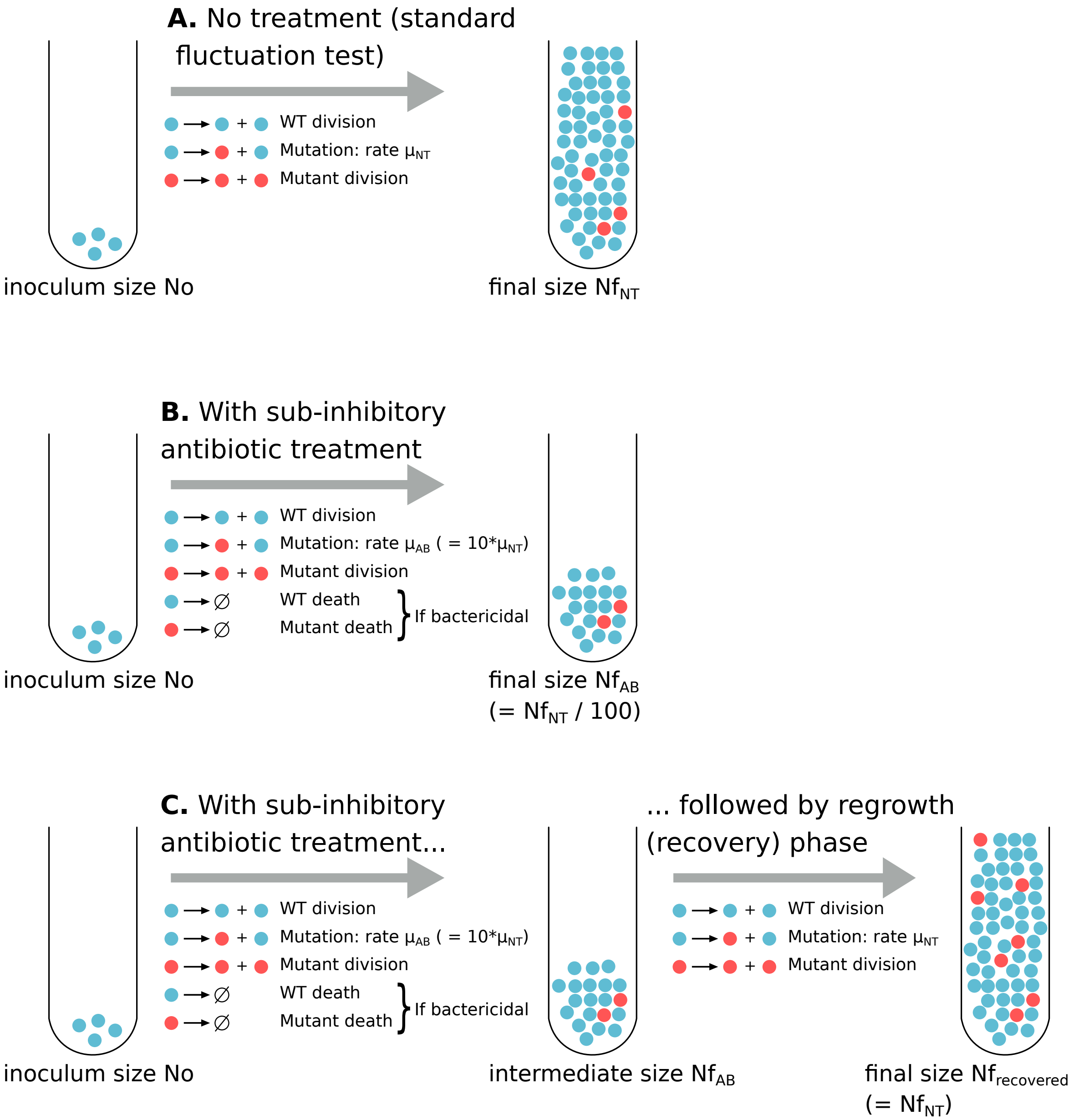

Figure S2: Simulated model and qualitative illustration. Two populations of individuals are considered: wild-type (WT, blue) and mutants for the trait of interest (red). Both can divide and potentially die. The mutation is neutral, and thus WT and mutants divide and die at the same rates. Backward mutation is neglected as fluctuation test only considers the situation where the mutant is rare. This model is simulated using Gillespie algorithm. On panel **A**, no treatment is applied (standard fluctuation test in the absence of stress). On panel **B**, a sub-inhibitory treatment is applied, increasing mutation rate due to mutagenic effects but decreasing reached population size due to bacteriostatic and / or bactericidal effects. On panel **C**, the same sub-inhibitory treatment is applied, but is followed by a regrowth phase in which the cells recover (and can keep dividing) in the absence of treatment.

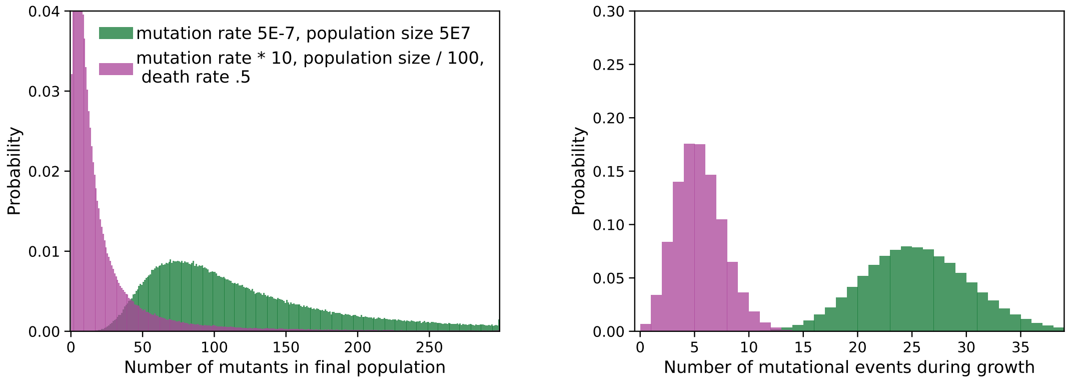

Figure S3: Change in genetic diversity due to a hypothetical treatment that increases mutation rate (x10) but decreases population size (/100), with death rate 0.5. This is similar to Fig. 1 of the main text, but with a treatment that has a bactericidal activity and not only a bacteriostatic activity.

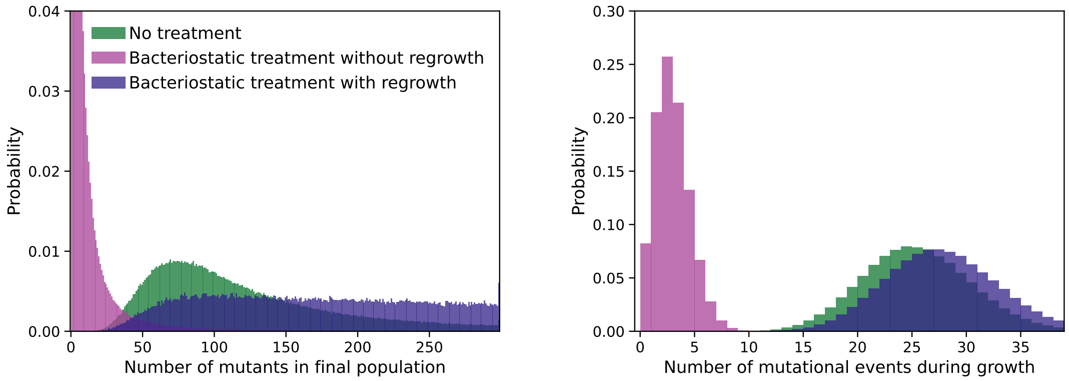

Figure S4: Change in genetic diversity with and without regrowth for a bacteriostatic treatment that increases mutation rate (x10) but decreases population size (/100). The data for untreated population and treatment without regrowth are the same as on Fig. 1 of the main text. The data for regrowth are produced by similar simulations, but with two phases: treatment (mutation rate x10) followed by recovery (mutation rate back to normal).

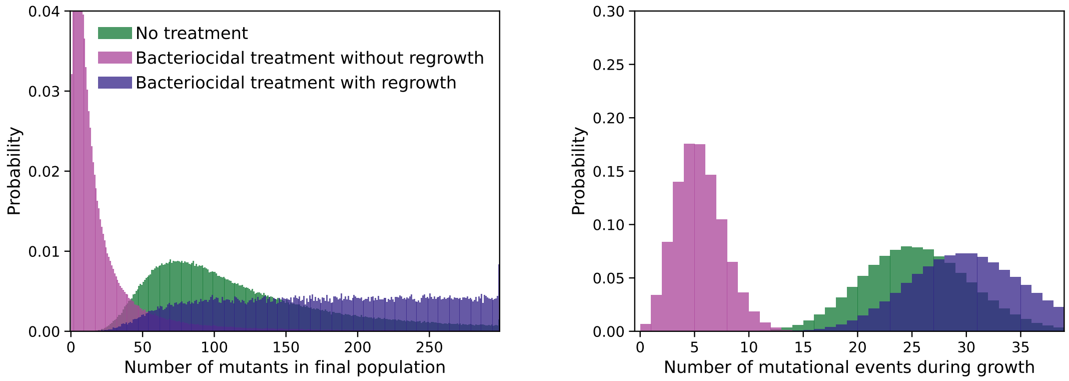

Figure S5: Change in genetic diversity with and without regrowth for a bactericidal treatment that increases mutation rate (x10) but decreases population size (/100) with death rate 0.5. This is similar to figure S4, but with a treatment that has a bactericidal activity and not only a bacteriostatic one. The relationship between the number of mutational events during growth and the number of mutants in the final population is more complex, because the lineage of a mutant may get extinct.

Author	Treatment	Protocol	Mutation rate	Population size	Mutants
Baharoglu	Ampicillin 0.05ug/mL	FT	2.59	0.64	0.87
Baharoglu	Ciprofloxacin 0.05ug/mL	FT	4.12	0.68	2.04
Baharoglu	Chloramphenicol 0.15ug/mL	FT	1.78	0.88	0.92
Baharoglu	Gentamicin 0.1ug/mL	FT	1.86	0.79	1.23
Baharoglu	Kanamycin 0.2ug/mL	FT	1.48	0.95	0.73
Baharoglu	Mitomycin C	FT	2.39	0.77	0.93
Baharoglu	Neomycin 0.1ug/mL	FT	2.40	0.81	1.04
Baharoglu	Rifampin 0.05ug/mL	FT	2.68	0.67	0.99
Baharoglu	Spectinomycin 0.2ug/mL	FT	2.31	0.73	1.18
Baharoglu	Tetracycline 0.15ug/mL	FT	2.66	0.94	1.53
Baharoglu	Tobramycin 0.1ug/mL	FT	1.21	0.73	0.50
Baharoglu	Trimethoprim 0.05ug/mL	FT	21.12	0.02	0.17
Cortes	Chloramphenicol 3.0ug/mL	FT	1.46	1.15	2.29
Cortes	Chloramphenicol 3.0ug/mL	FT	0.95	1.11	1.42
Cortes	Erythromycin 0.09ug/mL	Increase	0.78	1.30	1.29
Cortes	Erythromycin 0.09ug/mL	FT	1.25	0.98	1.03
Cortes	Penicillin 0.024ug/mL	FT	2.52	0.36	1.00
Cortes	Penicillin 0.024ug/mL	FT	0.97	0.34	0.23
Dapa	Mitomycin C 1ug/mL	FT	6.66	0.09	0.54
Dapa	Mitomycin C 1ug/mL	FT	6.46	0.09	0.58
Frenoy	Kanamycin 3ug/mL	FT	1.67	0.54	0.52
Frenoy	Kanamycin 3ug/mL	FT	0.69	0.24	0.10
Frenoy	Kanamycin 3ug/mL	FT	0.85	0.57	0.32
Frenoy	Kanamycin 3ug/mL	Increase	0.27	1.93	0.39
Frenoy	Norfloxacin 0.05ug/mL	FT	3.35	0.04	0.05
Frenoy	Norfloxacin 0.05ug/mL	FT	1.88	0.04	0.02
Frenoy	Norfloxacin 0.05ug/mL	FT	8.94	0.03	0.19
Frenoy	Norfloxacin 0.05ug/mL	FT	1.50	0.12	0.09
Frenoy	Norfloxacin 0.05ug/mL	FT	11.55	0.04	0.31
Frenoy	Norfloxacin 0.05ug/mL	FT	22.39	0.01	0.07
Giroux	Trimethoprim 0.04ug/mL	FT	5.47	0.45	2.99
Hocquet	Metronidazole 50ug/mL	FT	16.99	0.12	1.84
Hocquet	Metronidazole 50ug/mL	FT	4.08	0.43	2.25
Hocquet	Metronidazole 50ug/mL	Increase	3.51	1.70	2.82
Hocquet	Metronidazole 50ug/mL	FT	1.29	0.84	0.86
Jara	Ciprofloxacin 0.0625ug/mL	Regrowth	8.51	0.48	6.42
Jara	Colistin 2.5ug/mL	Regrowth	0.58	0.83	0.61
Jara	Meropenem 0.125ug/mL	Regrowth	0.55	0.43	0.18
Jara	Tetracycline 1.5ug/mL	Regrowth	2.40	0.45	1.33
Mo	Ampicillin 2ug/mL	Increase	1.97	1.49	3.40
Mo	Ampicillin 2ug/mL	Increase	5.07	4.81	62.20
Mo	Ciprofloxacin 0.01ug/mL	Increase	3.99	1.41	6.40
Mo	Ciprofloxacin 0.01ug/mL	FT	8.67	0.58	6.80
Mo	Mitomycin C 0.5ug/mL	FT	4.96	0.38	1.80
Mo	Mitomycin C 0.5ug/mL	FT	2.97	0.81	2.20
Mo	Nitrofurantoin 2ug/mL	FT	3.57	0.83	2.80
Mo	Nitrofurantoin 4ug/mL	Increase	9.25	4.32	54.00
Mo	Novobiocin 16ug/mL	Increase	1.29	2.52	3.60
Mo	Novobiocin 16ug/mL	Increase	1.77	3.48	8.60
Mo	Streptomycin 2ug/mL	FT	0.51	1.11	0.40
Mo	Streptomycin 2ug/mL	FT	1.31	0.85	0.80
Mo	Trimethoprim 0.032ug/mL	Increase	1.15	4.56	12.00
Mo	Trimethoprim 0.032ug/mL	Increase	4.69	2.89	26.60
Rodríguez-Rojas	Ampicillin 3.2ug/mL	Regrowth	3.56	0.73	2.83

Author	Treatment	Protocol	Mutation rate	Population size	Mutants
Rodríguez-Rojas	Ampicillin 3.2ug/mL	Regrowth	4.31	0.98	4.64
Rodríguez-Rojas	Ciprofloxacin 0.05ug/mL	Regrowth	4.02	0.73	3.87
Rodríguez-Rojas	Ciprofloxacin 0.05ug/mL	Regrowth	8.35	0.80	6.64
Rodríguez-Rojas	Kanamycin 1.6ug/mL	Regrowth	3.82	0.77	3.57
Rodríguez-Rojas	Kanamycin 1.6ug/mL	Regrowth	4.11	0.95	3.77
This Study	Ampicillin 1ug/mL	FT	0.96	1.17	0.90
This Study	Ampicillin 3.2ug/mL	FT	0.77	1.12	0.55
This Study	Ciprofloxacin 0.005ug/mL	FT	1.51	0.79	1.10
This Study	Chloramphenicol 0.15ug/mL	FT	0.85	1.03	0.70
This Study	Chloramphenicol 1.5ug/mL	FT	1.10	1.02	0.90
This Study	Kanamycin 1.6ug/mL	FT	0.31	0.26	0.00
This Study	Mitomycin C 1ug/mL	FT	61.01	0.00	0.00
This Study	Nalidixic acid 1ug/mL	FT	0.93	1.03	1.00
This Study	Norfloxacin 0.005ug/mL	FT	0.67	1.10	0.75
This Study	Norfloxacin 0.05ug/mL	FT	3.67	0.08	0.15
This Study	Streptomycin 5ug/mL	FT	1.09	0.11	0.00
This Study	Tetracycline 0.150ug/mL	FT	0.79	1.03	0.65
This Study	Trimethoprim 0.005ug/mL	FT	0.61	1.00	0.40
This Study	Trimethoprim 0.05ug/mL	FT	1.68	0.72	1.20
Torres Barceló	Ciprofloxacin 0.048ug/mL	FT	3.47	0.23	1.09
Torres Barceló	Ciprofloxacin 0.048ug/mL	FT	1.72	0.46	1.09
Torres Barceló	Ciprofloxacin 0.048ug/mL	FT	3.95	0.27	1.08
Torres Barceló	Ciprofloxacin 0.048ug/mL	FT	1.89	0.57	1.08

Table S6: In addition to the full dataset uploaded on Zenodo, we present here as a table the summary statistics for each experiment in our dataset used to produce figures 2 and 3 of the main text. Each data point represents one experiment (comprising several replicate populations) for each analyzed paper. Mutation rates, population sizes, and number of mutants are indicated as fold-change (compared to the untreated control) of the median of the replicates.
